# Prolonged Oxygen Therapy Post COVID-19 Infection: Factors Leading to the Risk of Poor Outcome

**DOI:** 10.7759/cureus.13357

**Published:** 2021-02-15

**Authors:** Alokananda Ray, Rajan Chaudhry, Sudhir Rai, Sujata Mitra, Sridhar Pradhan, Ashok Sunder, Deb Sanjay Nag

**Affiliations:** 1 Obstetrics and Gynaecology, Tata Main Hospital, Jamshedpur, IND; 2 Surgery, Tata Main Hospital, Jamshedpur, IND; 3 Medical Services, Tata Main Hospital, Jamshedpur, IND; 4 Nuclear Medicine, Tata Main Hospital, Jamshedpur, IND; 5 Indoor Medical Services, Tata Main Hospital, Jamshedpur, IND; 6 Internal Medicine, Tata Main Hospital, Jamshedpur, IND; 7 Anaesthesiology, Tata Main Hospital, Jamshedpur, IND

**Keywords:** covid-19, oxygen requirement, co-morbidities, risk factors

## Abstract

Background: Coronavirus disease 2019 (COVID-19) infection is caused by severe acute respiratory syndrome coronavirus 2 (SARS-CoV-2), a single-stranded ribonucleic acid (RNA) β-coronavirus. Prolonged duration of symptoms, ill health, disability, and need for hospitalisation are all well-known features of severe COVID-19 disease.

Objective: To describe the epidemiological, clinical and imaging characteristics of hospitalised patients of COVID-19 who required prolonged oxygen therapy after testing negative for SARS-CoV-2 and attempt to determine the associated factors leading to delayed recovery, failure to wean, and mortality.

Material and Method: Prospective observational study from 9th September to 6th November 2020 in a tertiary care COVID hospital of Jharkhand. Included COVID-19-infected patients requiring oxygen to maintain a saturation of ≥95% after testing reverse transcription polymerase chain reaction (RT-PCR) negative. Patients were classified as Group I, those who could be weaned off oxygen, and Group II, those who could not be weaned off oxygen during their stay in the isolation ward. A detailed assessment for outcome in these two groups related to age, gender, presence or absence of co-morbidities, nature of co-morbidities and findings of high-resolution CT (HRCT) thorax was done to ascertain risk factors for failure to wean and adverse outcomes.

Results: During the study period, 93 patients suffering from moderate to severe COVID-19 infection, could not be discharged from the hospital and were admitted to the post-COVID isolation ward after testing RT-PCR negative, due to breathlessness and need for oxygen therapy, with a male predominance, M:F ratio of 2.2:1. Of these 93 patients, 51 could be weaned off oxygen in the isolation ward. The mean and median age of patients who could be successfully weaned was 58.5±14.3 years and 60 years respectively, compared to a mean age of 64±12.4 years and a median age of 67 years for patients who could not be weaned off oxygen during the isolation period. Patients aged ≥60 years were at risk for prolonged requirement of oxygen compared to those <50 years of age, relative risk (RR) 1.43 (95%CI 0.9-2, p=0.051). Failure to wean in <50 years was noted in presence of co-morbidities, RR 4 (95%CI 1.5-10.6, p=0.005). Multivariable logistic regression analysis calculated an odds ratio (OR) of 12.22 (95%CI 2.4-61.5, p<0.002) in patients of coronary artery disease (CAD), and 3.34 (95%CI 1.01-10.9, p<0.046) in patients of diabetes, for failure to wean with delayed recovery in patients aged 50 years and more, having multiple co-morbidities. Presence of ≥three co-morbid conditions was associated with increased risk of critical care unit (CCU) admissions (RR 2.1, p=0.02), failure to wean (RR 1.79, p<0.006), and death (p=0.02). Elderly male patients (mean age of 81.3±7.2years) with CAD and multiple comorbidities were at a high risk of mortality (p=0.01).

Conclusion: Patients ≥50 years of age having ≥three co-morbidities are at increased risk of prolonged hospitalisation and oxygen therapy in moderate to severe COVID-19 infection, precluding their discharge even after they test negative for SARS-CoV-2. Elderly male patients of COVID-19 with CAD and multiple comorbidities are at a high risk of mortality.

## Introduction

Coronavirus disease 2019 (COVID-19) infection is caused by severe acute respiratory syndrome coronavirus 2 (SARS-CoV-2), which is a new, single-stranded ribonucleic acid (RNA) β-coronavirus [1]. Prolonged duration of symptoms, ill-health, disability, and need for hospitalisation are all well-known features of severe COVID-19 disease [2]. Understanding the factors that lead to poor outcome can enable health care professionals to appropriately tailor interventions, allocate resources, formulate hospital policies, and prognosticate patient outcomes.

The objective of this study was to describe the epidemiological, clinical and imaging characteristics of hospitalised patients with COVID-19 requiring prolonged oxygen therapy after testing reverse transcription polymerase chain reaction (RT-PCR) negative for SARS-CoV-2, and attempt to determine the associated factors leading to delayed recovery, failure to wean, and mortality.

## Materials and methods

The study was conducted in the post-COVID infection isolation ward (further referred to as isolation ward) of a tertiary care hospital with a COVID facility in the state of Jharkhand, India. This was a prospective observational study from 9th September to 6th November 2020. The sample size included all COVID-19-infected patients who were admitted to the isolation ward for breathlessness requiring oxygen to maintain a saturation of ≥95%, precluding their discharge. All of them had been admitted to the COVID facility of the hospital for at least 10 days from the onset of symptoms, and had tested RT-PCR negative for SARS-CoV-2, prior to transfer to the isolation ward. The study period corresponded to the time when the highest number of COVID-19-infected patients remained hospitalized for various indications, even after testing RT-PCR negative for SARS-CoV-2 (peak hospital admission rates due to active COVID-19 infection was from August to September 2020).

The discharge policy followed by the hospital was as per the state government directive for all hospitalised patients suffering from COVID-19 [3]. Patients with moderate and severe COVID-19 disease were discharged after 10 days of symptom onset, if symptoms resolved and there was no oxygen requirement for at least three consecutive days (moderate disease was defined as pneumonia with presence of clinical features of dyspnoea and or hypoxia, fever, cough, oxygen saturation 90-94% on room air, respiratory rate 15-30 breaths/minute; severe disease was defined as clinical signs of pneumonia with any of the following: respiratory rate >30 breaths/min, acute respiratory distress syndrome (ARDS), oxygen saturation <90% on room air, or septic shock). When the discharge criteria were not met, patients were stabilised and shifted to the isolation ward after they tested RT-PCR negative for SARS-CoV-2. These patients were further managed in the isolation ward for the stipulated isolation period of seven days (14 days prior to 14th September), till they either met the criteria for discharge, or were shifted out to non-COVID facilities of the hospital after completion of the isolation period. In the event of worsening symptoms during isolation, patients were transferred to the isolation critical care unit (CCU) for further management.

Outcome measures included successful weaning from oxygen, length of stay (LOS) and need for transfer to CCU, discharge, or death within the quarantine period in the isolation ward.

Based on requirement of oxygen support, patients were divided into two groups: Group I, those who could be weaned off oxygen, and Group II, those who could not be weaned off oxygen in the ward within the isolation period as mentioned above. A detailed assessment for outcome in these two groups related to age, gender, presence or absence of co-morbidities, nature of co-morbidities, and findings of high-resolution computerized tomography (HRCT) of thorax for severity score was done to ascertain high-risk factors for adverse outcome. For HRCT thorax, each lung lobe was evaluated by 0-5 points based on the area involved, with score 0 for normal performance, 1 for less than 5%, 2 for 6%-25%, 3 for 26%-50%, 4 for 51%-75%, and 5 for >75% of lung lobe areas involved. The total score was recorded by addition of the score of each lobe for a minimum of 0 and a maximum of 25. A score of <8 was reported as mild, 9-15 as moderate, and >15 as severe [4].

Continuous variables were summarised by their means and standard deviation (SD) and categorical variables were summarised by the corresponding counts and percentages. Risk analysis was done by calculating relative risk (RR), odds ratio (OR) and multivariate-regression analysis. Test of significance between groups was calculated by Chi square test or Fisher’s exact test. A p value of <0.05 was taken as significant.

## Results

The study group included 93 COVID-19 infected patients who were admitted in the isolation ward due to breathlessness needing oxygen therapy to maintain a saturation of ≥95% after testing negative for SARS-CoV-2, precluding their discharge.

During the study period from 9th September to 6th November 2020, a total of 156 patients could not be discharged from the COVID-positive facilities for various indications and were admitted to the isolation ward after they had tested negative for SARS-CoV-2. One hundred eight were males and 48 were females, with an M:F ratio of 2.2:1. The most common cause for prolonged hospitalisation in these patients was need for oxygen therapy to maintain a saturation of ≥95%, seen in 93 (59.6%) patients which constituted our study group. Of the 30/156 patients who were ≥70 years of age, 29 (96.6%) required oxygen at the time of transfer to the isolation ward. A detailed assessment of the 93 patients requiring oxygen, with respect to age, gender, presence of co-morbidities, and HRCT chest findings, was done to ascertain risk factors for poor outcome.

Sixty-four patients requiring oxygen therapy at transfer to the isolation ward were males and 29 were females (M:F ratio of 2.2:1). Eighteen (19.3%) patients were <50 years and 75 (80.6%) were ≥50 years of age (Table 1). Patients ≥60 years were at a higher risk for prolonged requirement of oxygen compared to those <50 years (RR 1.43, 95%CI 0.9-2, z statistic 1.9, p=0.051). Failure to wean off oxygen in the isolation ward for the same age group had a RR of 1.83 (95%CI 0.83-4.04, z statistic 1.5, p=0.13).

**Table 1:** Gender and age distribution with length of hospital stay in the isolation ward for the entire study population, Group I and Group II. N=93 SD: standard deviation, LOS: length of stay, ISOW: isolation ward. Group I: patients successfully weaned from oxygen in the isolation ward. Group II: patients who failed to be weaned from oxygen in the isolation ward.

Parameter	Total study population	Group I (Weaned from oxygen in ISOW)	Group II (Not Weaned off oxygen in ISOW)
Patients requiring Oxygen therapy	93	51	42
Male	64	37	27
Female	29	14	15
Age <50 (years)	18	13	5
Age ≥50 (years)	75	38	37
Age range (years)	25-86	26-82	25-86
Median Age (years)	62.5	60	67
Mean Age ± (SD) (years)	60.5 ± 13.7	58.5 ± 14.3	64 ± 12.4
Median LOS before transfer to ISOW (days)	14	11	17
LOS in ISOW Mean ± SD (days)	5.5 ± 2.4	4.6 ± 1.7	6.0 ± 3.1

Fifty-one (54.3%) patients could be weaned off oxygen successfully in the isolation ward (Group I). This constituted 14 of 29 (48.2%) females and 37 of 64 (57.8%) males. The requirement of oxygen at transfer ranged from 2 to 5 L/min in this group, none required non-invasive ventilation (NIV) and weaning was completed over a period of one to four days. Thus, more males could be successfully weaned off during the isolation period, however this was not statistically significant (p=0.39).

Age of patients successfully weaned ranged from 26-82 years (mean 58.5±14.3 years, median 60 years); 13 (25.5%) patients were <50 years. The hospital stays in this group prior to transfer to the isolation ward ranged between 10 to 23 days (median 11 days), with 13 patients being transferred from CCU. For the 45 (88.2%) patients in this group who could be discharged from the ward within the isolation period, the average LOS in the isolation ward was 4.6±1.6 days.

Forty patients in Group I had at least one co-morbid condition. Eighteen patients had one, 17 had two and five had ≥three co-morbidities (Table 2). The most common co-morbidity was Type II diabetes mellitus (25, 49%). Others included hypertension (24, 47%), both diabetes and hypertension (16, 31.6%), obesity (BMI>35kg/m2) (six, 11.7%), underlying lung disease (four, 7.8%) and pre-existing cardiovascular disease (CVD) (three, 5.8%). In patients <50 years, four out of 13 had co-morbid conditions, the most common being obesity (three out of four, 75%).

**Table 2:** Nature of co-morbid conditions in the entire study population, Group I and Group II. COPD: chronic obstructive pulmonary disease, ILD: interstitial lung disease, CVD: cardiovascular disease, CAD: coronary artery disease, DCMY: dilated cardiomyopathy, LVF: left ventricular failure, EF: ejection fraction, CKD: chronic kidney disease.

Nature of co-morbidity	Total (%)	Group I Weaned (% with morbidity)	Group II Not weaned (% with morbidity)
Diabetes mellitus	60 (64.5)	26 (43.3)	34 (56.6)
Hypertension	43 (46.2)	25 (58.1)	18 (41.9)
Diabetes+ Hypertension	31 (33.3)	16 (51.6)	15 (48.4)
Pre-existing lung disease	7 (7.5)	4 (57.1)	3 (42.9)
COPD	4	3	1
COPD + Pulmonary TB	1	1	0
ILD	2	0	2
CVD	14 (15)	3 (21.4)	11 (78.6)
CAD	11	2	9
DCMY	2	1 (EF<35%)	1 (EF<25%)
New Onset LVF	1	0	1
Morbid Obesity	8 (8.6)	6 (75)	2 (25)
Psychosis/ Bi-polar disorder	3 (3.2)	0	3 (100)
CKD	2 (2.1)	0	2 (100)

Seventeen patients in Group I underwent HRCT thorax, of them the severity score was reported as mild in six (35.3%), moderate in three (17.6%) and severe in seven (41%). One was reported as lung fibrosis with bronchiectasis which was possibly a sequela of COVID-19 infection in the recovery phase. One patient with mild score with pleural effusion and two with severe score could not be discharged from the isolation ward despite being weaned from oxygen.

None of the patients in Group I required CCU care after being transferred to or from the isolation ward. There was no mortality in Group I.

Forty-two (45%) patients could not be weaned off oxygen during their stay in the isolation ward (Group II). This constituted 15/29 (51.7%) females and 27/64 (42.1%) males. The oxygen requirement ranged between 3-8 L/min at the time of transfer to the isolation ward, eight patients required NIV during their stay and six had to be transferred to CCU within the isolation period due to increasing requirement of oxygen and failure to maintain saturation >90% despite maximum supportive care. Age of patients in Group II ranged from 25-86 years (mean 64±12.4 years, median 67 years). Thirty-seven of 42 (88.1%) were ≥50 years and five of 42 (11.9%) <50 years of age. The hospital stays prior to transfer to the isolation ward ranged between 10 to 30 days, a median of 17 days, with 12 patients being transferred from the CCU. None of the patients in this group could be discharged from the isolation ward, two patients opted for discharge against medical advice. Thirty-one patients (73.8%) were transferred to the non-COVID ward facilities of the hospital for further management after completion of the isolation period.

Forty-one (97.6%) patients in Group II had co-morbid conditions. Fifteen had one, 14 had two and 12 had ≥three comorbidities (Table 2). The most common co-morbidity was Type II diabetes mellitus (34, 80.9%). Others included hypertension (18, 42.8%), both diabetes and hypertension (15, 35.7%), pre-existing CVD (10, 23.8%), suspected myocarditis with left ventricular failure (LVF) (one, 2.3%), bipolar disorder on anti-psychotic drugs (three, 7.1%), pre-existing lung disease (three, 7.1%), obesity (BMI>35Kg/m2) (two, 4.7%), and chronic kidney disease (CKD) (two, 4.7%). In the age group <50 years all five had co-morbid conditions, the most common being diabetes (three out of five, 60%); other single co-morbid conditions included psychosis and obesity.

Sixteen patients (38%) in Group II had undergone HRCT thorax; the severity score was reported as mild in one (6.2%), moderate in four (25%), severe in 10 (62.5%) and one (6.2%) was reported as interstitial lung disease (ILD). Seven of the 10 cases with severe score needed CCU care, five before and two after transfer to the isolation ward, both of whom had a score of ≥22/25. None of the remaining 14 patients could be discharged from the isolation ward. The patient with mild score expired, she had multiple co-morbidities including CAD and dilated cardiomyopathy (DCMY).

There were three deaths in Group II. The age of these patients ranged between 73-86 years (mean 81.3±7.2 years), and LOS one to eight days (mean 3.3 days). All three had ≥three co-morbidities with CAD and Type II diabetes mellitus in common. The oxygen requirement was >5 L/minute in all, with one patient transferred from CCU requiring NIV. CAD with diabetes mellitus in the elderly patients was associated with a very high risk of mortality (RR 40, p<0.01).

The presence of co-morbidity co-related with adverse outcomes, as did the number of co-morbid conditions in this study. Increasing age was a compounding factor for presence of co-morbidities. Eighteen patients were aged <50 years, nine (50%) had no co-morbidities, all of them could be weaned off oxygen and discharged from the isolation ward with a mean LOS of 3.6±1.2 days. The risk of failure to wean for patients <50 years was significantly higher in presence of co-morbidities, compared to those without (RR 4, 95%CI 1.5-10.6, z statistic 2.7, p=0.005). The common morbidities for age <50 years were Type II diabetes (five of 18, 27.7%) and obesity (BMI>35Kg/m2) (four of 18, 22.2%). Type II diabetes, obesity, and psychosis were associated with inability to wean patients <50 years.

Seventeen patients across different age groups had ≥three co-morbid conditions, the risk of failure to wean from oxygen in them compared to patients with two or fewer co-morbidities was statistically significant (RR 1.79, 95%CI 1.1-2.7, z statistic 2.7, p=0.006). Eight of these patients required admission to critical care (RR 2.1, 95%CI 1.09-4.05, z statistic 2.2, p=0.02) and all deaths occurred in patients having ≥three morbidities (p=0.02). Thus, patients with three or more co-morbid conditions were at significantly higher risk of admission to the CCU, prolonged oxygen requirement, and death.

Type II diabetes mellitus was the most common co-morbid condition, present in 60/93 (64.5%) patients requiring oxygen therapy at the time of transfer to the isolation ward. The same was true for younger patients <50 years requiring prolonged hospital stay (five of 18, 27.7%). Multivariate regression analysis showed that patients with Type II diabetes in our study were 3.34 times more likely to have prolonged and higher oxygen requirement with failure to wean in patients >50 years with multiple co-morbid conditions (OR 3.34, p<0.046, 95%CI 1.01-10.9).

Fourteen of the 93 (14.9%) patients on oxygen therapy had CVD. Eleven of 42 (26.1%) patients who could not be weaned (Group II) had CVD (RR 2.03, 95%CI 1.38-2.98, z statistic 3.6, p<0.0003). CAD was present in 11 (78.5%) patients with CVD; the mean age of patients with CAD was 74.9±6.3 years which was 14.4 years more than the mean age of the study population, and 10 out of 11 cases of CAD (90.9%) were males. Multivariate regression analysis calculated an OR of 12.22 (95%CI 2.4-61.5, p<0.002) for failure to wean and delayed recovery in patients of CAD aged >50 years and having multiple co-morbidities. All three patients who expired in the isolation ward had CAD along with other co-morbid conditions (RR 40, 95%CI 4-742, z statistic 2.4, p=0.01). Thus, CAD appeared to be a very high-risk co-morbidity for patients infected with COVID-19.

Seven of 93 (7.5%) patients had pre-existing lung disease. The most common pre-existing lung condition was chronic obstructive pulmonary disease (COPD) in five, followed by interstitial lung disease (ILD) in two. Four patients had been transferred from the CCU (three cases of COPD and one case of ILD). One patient with COPD also had associated pulmonary tuberculosis and was on anti-tubercular treatment. Four cases of COPD (80%) could be weaned off oxygen. All four were successfully discharged from the isolation ward with a mean LOS of 3.7±0.95 days. One case of COPD and both cases of ILD requiring NIV could not be weaned. Thus, patients with ILD required longer duration of oxygen support compared to those with COPD.

HRCT chest was done in 33 patients in this study. Seven patients had mild, seven moderate and 17 had a severe score, one was reported as fibrosis with bronchiectasis which was probably a sequela of severe disease in the recovery phase and another one was reported as ILD. Increased morbidity was encountered in patients with severe score: 10/17 (58.8%) could not be weaned; these patients also required higher flow rates of oxygen including NIV compared to one out of seven (14.2%) with a mild score (p=0.13). Eleven patients with severe score (64.7%) required CCU admissions compared to one (14.2%) in the mild and two (28.4%) in the moderate score group (p=0.109).

## Discussion

In this study, we have attempted to classify the epidemiological and HRCT chest findings associated with prolonged requirement of oxygen therapy in 93 patients of moderate to severe COVID-19 disease and identify prognostic indicators for poor outcome. All patients had a median hospital stay of 14 days in the COVID positive facilities of the hospital prior to being included in the study on being transferred to the isolation ward after testing negative for SARS-CoV-2. Patients with a longer LOS in the COVID positive facilities prior to transfer were more difficult to wean and represented sicker patients with more severe disease.

High-risk factors for poor outcome and prognostic indicators for COVID-19 infection are poorly understood. In this study, a greater number of males (64, 68.8%) were affected compared to females (29, 31.2%), with an M:F ratio of 2.2:1. This gender distribution has also been observed in other studies for various Coronavirus infections [5]. The X chromosome and female sex hormones have been implicated to play an important role in innate immunity against SARS-CoV-2 in females [6]. The effect of gender on clinical outcome in patients of COVID-19 remains controversial [7,8]. In our study, more males could be successfully weaned off during the isolation period compared to females, however this was not statistically significant (p=0.39). Delayed recovery and return to normal health has been reported amongst hospitalised patients suffering from COVID-19 aged >50 years with co-morbidities compared to the younger age group or those having no co-morbid conditions [2]. A study reported high proportion of severe to critical disease and rapid progress leading to death after admission in patients aged >60 years with dyspnoea; ARDS, CAD, and COPD were strong predictors of death [9].

The mean age of patients with poor outcome in our study was greater by 6±2.1 years and the median age seven years older than that of the patients with favourable outcome. Age ≥60 years was associated with prolonged hospitalisation and oxygen requirement with a RR of 1.43 (95%CI 0.9-2, z statistic 1.9, p=0.051) compared to those <50 years of age. All three patients who died in our study were >70 years old (mean 81.3±7.2 years) and had multiple co-morbid conditions. The risk of failure to wean from oxygen and delayed recovery in young patients <50 years was significantly higher in presence of co-morbidities compared to those who had none in this age group (RR 4, 95%CI 1.5-10.6, z statistic 2.7, p=0.005). Diabetes, obesity, and psychosis were associated with inability to wean patients <50 years of age.

Type II diabetes mellitus was the most common co-morbid condition, present in 60/93 (64.5%) patients requiring oxygen therapy at the time of transfer to the isolation ward. The same was true for younger patients <50 years requiring prolonged hospital stay (five of 18, 27.7%). Patients with diabetes mellitus are known to have severe COVID-19 infection with adverse outcome; the infection itself can hinder effective glycaemic control, necessitating close monitoring and careful management in diabetic patients [10]. Several studies have found an association between diabetes and ARDS, CCU admission, and death in patients with COVID-19 [11,12]. Diabetes has been listed in the top three co-morbid conditions with a total co-morbidity prevalence as high as 78% among intensive care unit (ICU) COVID-19 admissions [13].

Multivariate logistic analysis showed that patients with Type II diabetes in our study were 3.34 times more likely to have prolonged and higher oxygen requirement with failure to wean in patients >50 years with multiple co-morbid conditions (OR 3.34, p<0.046, 95%CI 1.01-10.9). Collectively several studies across the globe have suggested that patients with diabetes have an increased likelihood of adverse outcome including death, however other existing risk factors may contribute to variable conclusions [14]. The French multicentre study CORONADO found no difference in outcome with Type I and Type II diabetes and COVID-19 infection, however the UK National Health Service (NHS) study concluded that patients with Type I diabetes are at a greater risk for adverse outcome [15,16]. None of the patients in our study had Type I diabetes mellitus.

Other co-morbid conditions reported in various studies as risk factors for hospitalisation and severe COVID-19 infection include hypertension, CAD, CKD, and obesity [17,18]. Hypertension was encountered in 43/93 (46.2%) and was the second most prevalent co-morbid condition in our study. Thirty-one patients (33%) had both diabetes and hypertension.

The prevalence of CAD in COVID-19 infection has been reported as 2.5 to 10% in various studies [19,20]. In our study, it was 11/93 (11.8%). The average age of patients with CAD is known to be older with a male predominance [21]. Ninety percent of the patients with CAD in our study were males and their mean age was 14.4±7.3 years older than that of patients without CAD. Several studies have reported an increased risk of ARDS, CCU admission, need for ventilation, and death in patients with CAD with COVID-19 infection, however it is not very clear to what extent the increased risk is attributable to CAD as an independent risk factor [20,21]. The patients with CAD in our study had significantly higher risk of adverse outcome with respect to prolonged hospital admission, oxygen therapy, CCU admission, and death; multivariable regression analysis calculated an OR of 12.22 (95%CI 2.4-61.5, p<0.002) for failure to wean and delayed recovery in patients with CAD aged >50 years having multiple co-morbidities. All three deaths in our study occurred in patients with CAD, they were all >70 years of age and had ≥three co-morbid conditions. In a study from Italy, the unadjusted probability of death in the patients with CAD was 0.49 compared to 0.20 in those without CAD. However, after multivariable analysis and adjusted hazard ratio (aHR), the study concluded that although patients with CAD with COVID-19 infection have a very high risk of mortality, it can be attributed to the burden of other co-morbidities rather than due to CAD per se [21]. The two variables which were independently associated with all-cause mortality in their study were advanced age (added to the risk) and female gender (reduced the risk) [21].

A French cohort found obesity (BMI >35kg/m2) to be a strong predictor of need for ventilation; the multivariate odds ratio was 7.36 after correction for age, sex, diabetes, and hypertension [22]. The Open-SAFELY study also reported that mortality risk increased with BMI, from HR 1.40 for class II obesity (BMI 35-39.9kg/m2) to 1.92 for class III obesity (BMI ≥40kg/m2) [23]. Studies have reported that increase in BMI proportionately increases hospitalization risk [24] and obesity to be an independent predictor of serious infection (multivariate OR 3.0) [25]. Eight patients in our study had a BMI of >35kg/m2, of whom five had required CCU admission and two could not be weaned off oxygen in the isolation ward. Obesity emerged as an important risk factor for CCU admission (RR 2.6, 95%CI 1.3-5.1, p<0.003) and an important co-morbidity in patients <50 years of age requiring prolonged hospitalisation and oxygen therapy (four of 18, 22.2%).

Given the devastating effect the COVID-19 infection has on the lung tissue, there is reason to fear the outcome of this infection in patients with underlying chronic lung disease. The prevalence of COPD in hospitalised COVID-19 patients ranges from 0 to 14% in various studies and is reported to be associated with severe infection [26]. A risk analysis for adverse outcome in 1590 COVID-19 patients across China reported an OR of 2.681 (95%CI 1.424-5.048, p=0.002) for ICU admission, mechanical ventilation, or death after adjustment for age and smoking in patients with COPD [27]. An international multicentre study on outcome of COVID-19 infection in hospitalised patients with ILD concluded that these patients (especially fibrotic ILD) had a higher risk of mortality. The risk increased with age, male gender, obesity, and pre-infection poor lung function in the range of moderate to severe disease [28]. In our study, seven (7.5%) patients had underlying chronic lung disease, with COPD being more prevalent in five (5.3%) and ILD in two (2.1%). One patient with COPD and both patients with ILD could not be weaned off oxygen in the isolation ward. Patients with ILD required a longer duration of hospitalisation and oxygen support with failure to wean compared to those with COPD.

COVID-19 infection with more than one co-morbid condition makes the patient vulnerable to increased risk of morbidity and mortality. In a study from New York City, 88% of hospitalised patients had ≥two co-morbidities compared to 6.3% with one [29]. A scoring system to predict adverse outcome in patients of COVID-19 found the number of co-morbidities (OR 1.6) as one of the 10 important variables for risk assessment [30]. Multiple co-morbid conditions co-related with poor outcome in our study. Three or more co-morbidities were associated with increased risk of CCU admissions (RR 2.1, p=0.02), failure to wean with prolonged oxygen requirement (RR 1.79, p<0.006), and death (p=0.02).

Limitations

Our study had some limitations; it was a single centre study with small sample size. The study population only included COVID-19 patients who could not be discharged and were transferred to the isolation ward after testing negative for SARS-CoV-2. Study observation was limited to the period of stay in the isolation ward, lacking further follow up on outcome once transferred or discharged from the ward. Advice for HRCT thorax was not a defined management protocol during the study period and hence was not available for all patients. 

## Conclusions

In our study, we found that patients with COVID-19 who remained hospitalised after testing negative for SARS-CoV-2 were twice more likely to be males than females. Patients ≥60 years were at higher risk for prolonged oxygen requirement compared to younger patients <50 years, however this was not statistically significant. Patients with ≥three co-morbidities were at significant risk of CCU admissions, delayed recovery, and death. Type II diabetes mellitus, CAD, ILD, and obesity (BMI >35kg/m2) were important risk factors for failure to wean and delayed recovery in patients of COVID-19 who were ≥50 years of age. In younger patients <50 years, diabetes mellitus, obesity, and psychosis requiring anti-psychotic drugs were risk factors for delayed recovery.
